# Meteorological Register

**Published:** 1834-10-01

**Authors:** 


### METEOROLOGICAL REGISTER,

FROM JUNE 1 TO AUGUST 31.

By Messrs. HARRIS and Co., Mathematical Instrument Makers, 50, High Holborn.

| | Thermometer. | | Barometer. | | De Luc's Hygrometer. | | Winds. | | Atmospheric Variations. | | |
|---|---|---|---|---|---|---|---|---|---|---|---|
| 1834. | *max.* | *min.* | *max.* | *min.* | *max.* | *min.* | | | 9 *a. m.* | 2 *p.m.* | 10 *p.m.* |
| June 1 to 7 | 75 | 45 | 30.04 | 29.48 | 61 | 53 | NNE | SSW | fine | cloudy | rain |
| 14 | 74 | 51 | 29.65 | .41 | 70 | 58 | SSW | SE | fine | cloudy | rain |
| 21 | 84 | 54 | .86 | .42 | 70 | 60 | SSW | WSW | fine | rain | fine |
| 30 | 74 | 53 | 30.95 | .79 | 64 | 59 | WSW | SW | cloudy | fine | cloudy |
| July 1 to 7 | 75 | 57 | .97 | .63 | 85 | 65 | NE | SSW | cloudy | rain | cloudy |
| 14 | 76 | 58 | .82 | .58 | 79 | 62 | SSW | SW | showery | cloudy | fine |
| 21 | 82 | 58 | .94 | .40 | 90 | 62 | WSW | SE | fine | cloudy | rain |
| 31 | 78 | 55 | .75 | .52 | 98 | 65 | E | ESE | cloudy | fine | rain |
| Aug. 1 to 7 | 74 | 59 | .67 | .58 | 82 | 72 | S | N | fine | cloudy | rain |
| 14 | 78 | 58 | .82 | .75 | 82 | 64 | SW | S | fine | fine | fine |
| 21 | 74 | 62 | .92 | .48 | 72 | 65 | NNE | NW | fine | fine | fine |
| 30 | 67 | 49 | .65 | .37 | 77 | 63 | SSE | S | cloudy | fine | rain |

The quantity of Rain fallen in June, 1 inch and 33-100ths.
— — — July, 3 inches and 46-100ths.
— — — August, 1 inch and 96-100ths.

